# Letter to the editor: Towards effective implementation of hospital-onset bacteraemia and fungaemia as a quality metric

**DOI:** 10.2807/1560-7917.ES.2025.30.38.2500659

**Published:** 2025-09-25

**Authors:** Silvia Forni, Guglielmo Arzilli, Fabrizio Gemmi, Agnese Comelli, Gabriele Del Castillo, Simone Villa, Federico Banchelli, Enrico Ricchizzi, Fortunato D’Ancona

**Affiliations:** 1 Regional Health Agency of Tuscany, Florence, Italy; 2 Department of Translational Research and New Technologies in Medicine and Surgery, University of Pisa, Pisa, Italy; 3 Directorate General for Health, Lombardy Region, Milan, Italy; 4 Directorate General for Personal Care, Health and Welfare, Bologna, Italy; 5 Department of Infectious Diseases, Istituto Superiore Di Sanità, Rome, Italy

**Keywords:** hospital-onset bacteraemia and fungaemia, HOB, automated surveillance, electronic health records, bloodstream infection, indicator

**To the editor: **We read with great interest the article by Aghdassi et al. [1], which represents a notable step forward in the development of automated surveillance for hospital-onset bacteraemia and fungaemia (HOB). While recognising its value for surveillance, we would like to suggest how to improve the use of the HOB rate as a key performance indicator for measuring quality of care. 

Firstly, misclassification in HOB estimates could arise from different data collection protocols and diagnostic stewardship. As noted by Aghdassi et al. [1], adopting data structures that support interoperability across institutions is essential for automated surveillance. This requires large-scale standardised protocols for real-time microbiological data collection, based on common definitions and aligned with international standards, which can be integrated with other electronic health records (EHRs). In addition, a combined monitoring approach, incorporating the incidence of HOB, the number of blood cultures performed and the number of bloodstream infections sustained by common commensals, offers a more robust framework for developing and implementing strategies to reduce HOB. Moreover, HOB incidence is closely linked to the characteristics and organisation of the hospitals [2]. Aghdassi et al. showed that, within a hospital network, patient movements were classified as transfers rather than repeated admissions, considering the time spent at each facility and allowing for longer patient monitoring to better assess the overall HOB incidence across the network [1]. Drawing on this observation, incorporating information on inter-hospital transfers or readmissions would help mitigate the risk of misclassification in the estimation of HOBs [3].

Secondly, the usefulness of the HOB indicator is closely linked to preventability of HOBs. In the Discussion section, the authors highlight the need for future research aimed at characterising ‘*the conditions underlying HOB episodes, assess preventability and effective interventions*’ [1]. Leekha et al. estimate that 36% of non-commensal HOBs are preventable [4], effectively opening opportunities for improvement in prevention, in line with what has been observed for widely used quality and safety indicators, such as in-hospital mortality or readmissions [5]. However, this aspect raises a new issue related to the extent of human and financial resources needed to analyse cases and develop an improvement plan. Since the proportion of preventable cases may be low, especially in high-risk patients, a substantial number of cases should be reviewed to identify potentially preventable infections. This challenge is particularly relevant when compared with the central line–associated bloodstream infections (CLABSI) indicator, where the proportion of preventable infections is higher and the workload for infection prevention staff to analyse cases and develop an improvement plan is lower than for HOBs. One strategy to address this problem is to exclude from the indicator patients with high-risk conditions, e.g. neutropenia, immunosuppression [6].

Finally, specific attention should be paid to the intended purpose of using this indicator. Aghdassi et al. emphasise that this indicator can detect temporary increases in the same hospital such as those observed during the COVID-19 pandemic between 2020 and 2021 [1], highlighting potential vulnerabilities in the quality of care and patient safety [3]. However, in different contexts, such as value-based programs or for benchmarking, risk adjustment methods must be used to assess differences in the patients’ case mix across hospitals [6].

In relation to the above aspects, three Italian regions (Tuscany, Emilia-Romagna and Lombardy) are participating in a project coordinated by the National Institute of Health (ISS), within the European initiative ‘Surveillance from Electronic Health Data’ (SUREHD) funded by the European Centre for Disease Prevention and Control (ECDC) [7], which aims to define and test a shared protocol for the surveillance of bloodstream infections based on EHRs in European Union/European Economic Area (EU/EEA) countries.

In response to some of the points raised, the Italian SUREHD project plans to: (i) develop a hospital-level algorithm to estimate HOB incidence, define priorities, and track progress, without inter-hospital benchmarking; (ii) monitor blood culture rates and possibly restrict surveillance to pathogens with reliable data and established diagnostics; (iii) include most regional public hospitals, accounting for transfers and readmissions to reduce bias in defining HOB.

In conclusion, we agree with Aghdassi et al. that the introduction of automated HOB surveillance could represent a remarkable advancement in infection prevention and control. To maximise its effectiveness as a quality and safety indicator, it is essential to address additional challenges related to the accurate identification of bloodstream infections, evaluate their preventability and potential intervention strategies and clearly define its intended purpose, i.e. benchmarking, quality improvement or research.
